# Matching-adjusted indirect treatment comparison of chimeric antigen receptor T-cell therapies for third-line or later treatment of relapsed or refractory large B-cell lymphoma: lisocabtagene maraleucel versus tisagenlecleucel

**DOI:** 10.1186/s40164-022-00268-z

**Published:** 2022-03-25

**Authors:** Guillaume Cartron, Christopher P. Fox, Fei Fei Liu, Ana Kostic, Jens Hasskarl, Daniel Li, Ashley Bonner, Yixie Zhang, David G. Maloney, John Kuruvilla

**Affiliations:** 1 Montpellier University Hospital Center, 80 Avenue Augustin Fliche, Montpellier, France; 2 Nottingham University Hospitals NHS Trust, Nottingham, UK; 3 Bristol Myers Squibb, Princeton, NJ USA; 4 Bristol Myers Squibb, Seattle, WA USA; 5 Present Address: Celgene, a Bristol-Myers Squibb Company, Boudry, Switzerland; 6 EVERSANA, Burlington, ON Canada; 7 Fred Hutchinson Cancer Research Center, Seattle, WA USA; 8 Princess Margaret Cancer Centre, Toronto, ON Canada

**Keywords:** CAR T-cell therapy, Lisocabtagene maraleucel, Tisagenlecleucel, Indirect treatment comparison, Matching-adjusted indirect comparison

## Abstract

**Background:**

There are no head-to-head clinical studies comparing chimeric antigen receptor (CAR) T-cell therapies for the treatment of relapsed or refractory aggressive large B-cell lymphomas. Naive, indirect comparisons may be inappropriate, as the study designs and patient populations could differ substantially. Matching-adjusted indirect comparisons (MAIC) can reduce many biases associated with indirect comparisons between studies. To determine the comparative efficacy and safety of lisocabtagene maraleucel (liso-cel) to tisagenlecleucel, we describe an unanchored MAIC of the pivotal studies TRANSCEND NHL 001 (TRANSCEND; NCT02631044; liso-cel) and JULIET (NCT02445248; tisagenlecleucel).

**Methods:**

Individual patient data (IPD) from TRANSCEND were available to the authors; for the JULIET pivotal study, summary-level data from the published study were used. To balance the populations between two studies, IPD from TRANSCEND were adjusted to match the marginal distribution (e.g., mean, variance) of clinical factors among patients from JULIET.

**Results:**

Results from the primary MAIC showed liso-cel had statistically significant greater efficacy than tisagenlecleucel (objective response rate: odds ratio [OR] = 2.78, 95% confidence interval [CI]: 1.63‒4.74; complete response rate: OR = 2.01, 95% CI: 1.22‒3.30; progression-free survival: hazard ratio [HR] = 0.65, 95% CI: 0.47‒0.91; overall survival: HR = 0.67, 95% CI: 0.47‒0.95). MAIC of safety outcomes showed lower ORs for all-grade and grade ≥ 3 cytokine release syndrome, and grade ≥ 3 prolonged cytopenia for liso-cel when compared with tisagenlecleucel; there were no statistically significant differences detected for other safety outcomes.

**Conclusions:**

Overall, this MAIC of two CAR T-cell therapies indicates liso-cel had favorable efficacy and a comparable or better safety profile relative to tisagenlecleucel.

*Clinical trial registration*: ClinicalTrials.gov identifiers: NCT02631044 and NCT02445248.

**Supplementary Information:**

The online version contains supplementary material available at 10.1186/s40164-022-00268-z.

## Introduction

Non-Hodgkin lymphoma (NHL) is one of the most common types of cancer worldwide, with reported incidence rates of 6.7 per 100,000 in men and 4.7 per 100,000 in women in 2018 [1]. Diffuse large B-cell lymphoma (DLBCL) represents the most common NHL subtype, accounting for 30‒58% of NHL cases in Europe and 25% of cases in the United States [2, 3]. Between 2011 and 2012, the annual age-adjusted incidence rate of DLBCL was 3.8 per 100,000 persons in Europe and 6.9 per 100,000 persons in the United States [3, 4]. DLBCL can occur as de novo disease or arise as a transformation from other indolent forms of NHL. Treatment options for patients with relapsed or refractory (R/R) DLBCL are limited. These patients often receive salvage chemotherapies that confer poor survival outcomes; 4-year overall survival (OS) rate of 28% and median OS of 6 months in refractory patients [5].

Chimeric antigen receptor (CAR) T-cell therapies have shown clinical activity in patients with R/R large B-cell lymphoma, with objective response rates (ORR) and complete response (CR) rates ranging from 52 to 82% and from 40 to 54%, respectively [6–8]. Tisagenlecleucel, axicabtagene ciloleucel (axi-cel), and most recently, lisocabtagene maraleucel (liso-cel) have been approved in the United States for third-line or later treatment of large B-cell lymphoma (LBCL). While tisagenlecleucel and liso-cel utilize an anti-CD19 antigen-binding domain fused with the costimulatory 4-1BB and CD3ζ domains, the former has a CD8 hinge and transmembrane region, whereas the latter has an immunoglobulin G4 hinge region and CD28 transmembrane domain. Axi-cel utilizes an anti-CD19 antigen-binding domain fused to CD28 and CD3ζ costimulatory domains [9–11]. All three are single-dose products administered intravenously, though liso-cel has a defined composition of equal CD8^+^ and CD4^+^ cells with low variability. Dose and ratio of CD8^+^ and CD4^+^ CAR^+^ T cells may influence the incidence and severity of cytokine release syndrome (CRS) and neurological events (NE) [12–14]. It is unclear if the differences of these products affect clinical outcomes.

TRANSCEND NHL 001 (TRANSCEND; NCT02631044) was a phase 1, single-arm, multicenter, open-label study that sought to investigate the efficacy and safety of liso-cel as a treatment in patients with LBCL who have R/R disease after receiving at least two prior lines of therapy.[6] Patients with DLBCL not otherwise specified (de novo, transformed follicular lymphoma, and transformed indolent NHL), high-grade lymphoma with rearrangements in *MYC* and either *BCL2, BCL6,* or both, primary mediastinal B-cell lymphoma, and follicular lymphoma grade 3B were eligible if they had R/R positron emission tomography–positive disease after at least two lines of prior systemic therapy, including a CD20-targeted agent and anthracycline; had an Eastern Cooperative Oncology Group performance status (ECOG PS) of 0–2; and adequate organ function. Patients with secondary central nervous system (CNS) lymphoma or prior autologous or allogeneic hematopoietic stem cell transplantation (auto-HSCT or allo-HSCT, respectively) were permitted. However, patients with primary CNS lymphoma or allo-HSCT within 90 days of leukapheresis were excluded. Primary endpoints were adverse events (AE), dose-limiting toxicities, and ORR, as assessed by an independent review committee (IRC) per Lugano 2014 criteria [15]. Secondary endpoints included CR rate as assessed by IRC, duration of response, progression-free survival (PFS), and OS.

JULIET (NCT02445248) was a phase 2, single-arm, multicenter, open-label, registrational study of the efficacy and safety of tisagenlecleucel in patients with R/R LBCL [8]. Eligible patients had DLBCL, high-grade lymphoma with *MYC* rearrangement plus rearrangement of *BCL2*, *BCL6*, or both, or transformed follicular lymphoma; received at least two prior lines of therapy, including rituximab and an anthracycline; and were ineligible for or had disease progression after auto-HSCT. Patients were excluded if they had primary mediastinal B-cell lymphoma, had previously received allo-HSCT, or had secondary CNS lymphoma. The primary endpoint was best ORR, as assessed by IRC per Lugano 2014 criteria [15], and key secondary endpoints included duration of response, OS, and safety. CRS was originally graded according to the University of Pennsylvania criteria, but a secondary analysis aligned to the Lee 2014 criteria, which was used for this comparative analysis [16]. There are no head-to-head clinical studies comparing the CAR T-cell therapies to inform treatment decisions, policy decision-making, and other health care–related issues. Naive, indirect comparisons may be inappropriate, as the study designs and patient populations could differ substantially. Comparing interventions using matching-adjusted indirect comparison (MAIC) analyses can reduce many biases associated with indirect comparisons between studies by adjusting for differences in patient and study characteristics [17]. MAICs are increasingly being included in submissions to regulators and/or health technology assessment agencies. To determine the comparative efficacy and safety of liso-cel versus tisagenlecleucel, we describe a MAIC analysis of the pivotal studies TRANSCEND (liso-cel) and JULIET (tisagenlecleucel).

## Methods

### Data sources and study characteristics

MAIC methodology was used to estimate population-adjusted relative treatment effects associated with liso-cel compared with tisagenlecleucel. Table 1 summarizes the data sets used, and Table 2 lists study design characteristics and eligibility criteria for TRANSCEND and JULIET.

**Table 1:** Summary of datasets

Treatment	Study name	Data cutoff^a^ (MM/DD/YYYY)	Median study follow-up, months (range)	Analysis set	N
Efficacy outcomes					
Liso-cel	TRANSCEND [6]	08/12/2019	11.5 (0.2‒35.0)^b^	DLBCL efficacy set	256
Tisagenlecleucel—ORR, CR rate	JULIET [8]	12/08/2017	14 (0.1‒26)^c^	Efficacy analysis set	93
Tisagenlecleucel—PFS, OS	JULIET [8]	12/08/2017	14 (0.1‒26)^b^	Safety set/full analysis set	111
Safety outcomes					
Liso-cel	TRANSCEND [6]	08/12/2019	11.5 (0.2‒35.0)^b^	DLBCL-treated set	269
Tisagenlecleucel	JULIET [8]	12/08/2017	14 (0.1‒26)^c^	Safety set/full analysis set	111

Study	Data sources				
TRANSCEND	Individual patient data				
JULIET	Schuster et al. [8] was supplemented with the EMA Public Assessment Report [33], the EMA Summary of Product Characteristics [34], the United States FDA Summary Basis for Regulatory Action [35], and Schuster et al. [16]^d^				

*CR* complete response, *CRS* cytokine release syndrome, *DLBCL* diffuse large B-cell lymphoma, *EOS* end of study, *EMA* European Medicines Agency, *FDA* Food and Drug Administration, *liso-cel* lisocabtagene maraleucel, *ORR* objective response rate, *OS* overall survival, *PFS* progression-free survival

^a^Data cutoffs with most complete data availability were included

^b^Median on-study follow-up time was reported, which was defined as (EOS date—first dose date + 1)/30.4375. If patients were continuing on study, the data cutoff date was used to impute the EOS date for the purpose of the calculation

^c^Median follow-up time from infusion to data cutoff was reported [8]

^d^In JULIET, CRS was rated according to the University of Pennsylvania criteria. However, the JULIET investigators regraded CRS events according to the Lee 2014 criteria [36]; rates of CRS associated with tisagenlecleucel were extracted from Schuster et al. [16], which was based on the Lee 2014 criteria [36] and also used in TRANSCEND

**Table 2:** Study design characteristics and eligibility criteria for TRANSCEND and JULIET

	TRANSCEND (liso-cel) [6]	JULIET (tisagenlecleucel) [8]	
Key study design features			
Phase	1	2	
Design	Single arm	Single arm	
Blinding	Open label	Open label	
Centers	Multicenter	Multicenter	
Country	US	Multiple (US, Canada, Europe, Japan)	
Bridging therapy	Allowed	Allowed	
PET-positive disease after bridging therapy	Confirmed	Not always confirmed	
Lymphodepleting chemotherapy	Yes	Yes (omitted if white blood cell count ≤ 1000 cells/μL)	
Regimen and dosage of lymphodepleting chemotherapy	Fludarabine (30 mg/m^2^/day for 3 days) and cyclophosphamide (300 mg/m^2^/day for 3 days), completed 2‒7 days before infusion	Fludarabine (25 mg/m^2^ IV daily for 3 days) and cyclophosphamide (250 mg/m^2^ IV daily for 3 days, starting with the first dose of fludarabine) within 1 week before infusion Alternatively, bendamustine 90 mg/m^2^ IV daily for 2 days^a^	
CAR T-cell regimen and dosage	Dose level 1, single-dose regimen: 50 × 10^6^ CAR^+^ T cells (25 × 10^6^ CD8^+^ CAR^+^ T cells and 25 × 10^6^ CD4^+^ CAR^+^ T cells) Dose level 1, two-dose regimen: 50 × 10^6^ CAR^+^ T cells Dose level 2, single-dose regimen: 100 × 10^6^ CAR^+^ T cells (50 × 10^6^ CD8^+^ CAR^+^ T cells and 50 × 10^6^ CD4^+^ CAR^+^ T cells) Dose level 3, single-dose regimen: 150 × 10^6^ CAR^+^ T cells (75 × 10^6^ CD8^+^ CAR^+^ T cells and 75 × 10^6^ CD4^+^ CAR^+^ T cells)	Single infusion of 1 to 5 × 10^8^ CAR^+^ T cells	

	TRANSCEND (liso-cel) [6]	JULIET (tisagenlecleucel) [8]	Action taken for TRANSCEND IPD and rationale
Key inclusion criteria			
NHL subtype	DLBCL NOS, HGBCL, tFL, tiNHL, PMBCL, FL3B	DLBCL NOS, HGBCL, tFL	Recategorized TRANSCEND and JULIET to improve comparability of patients with DLBCL. For TRANSCEND, DLBCL NOS, HGBCL, and tiNHL were combined as “DLBCL”. For JULIET, DLBCL NOS, HGBCL, and other were combined into “DLBCL”
Age	≥ 18 years	≥ 18 years	None
ECOG PS	≤ 2^b^	≤ 1	None
Prior lines of treatment	≥ 2	≥ 2	Redefined in TRANSCEND such that a line of therapy included chemotherapy, auto-HSCT, allo-HSCT, and radiotherapy to align with JULIET definition
Prior auto-HSCT	Allowed	Allowed	None
Prior regimen required	Anthracycline and rituximab (or other CD20-targeted agents)	Included rituximab and anthracycline	None
Response to prior therapy	R/R disease after ≥ 2 lines of therapy or after auto-HSCT	R/R disease after ≥ 2 lines of chemotherapy, including rituximab and anthracycline Patients had to have either failed auto-HSCT, be ineligible for, or not consent to auto-HSCT	None
Absolute lymphocyte count	No minimum requirement^c^	≥ 300/μL	Redefined in TRANSCEND to align with JULIET definition
Absolute neutrophil count	No minimum requirement^c^	> 1000/μL	None
Platelet count	No minimum requirement^c^	≥ 50,000/μL	None
Hemoglobin	No minimum requirement^c^	> 8.0 g/dL	None
Alanine aminotransferase	≤ 5 × ULN	≤ 5 × ULN for age	None
Total bilirubin	< 2.0 mg/dL	≤ 2.0 × ULN	None
Serum creatinine	≤ 1.5 × ULN	≤ 1.5 × ULN	None
CrCl	> 30 mL/min/1.73 m^2^ (Cockcroft-Gault)	≥ 60 mL/min/1.73 m^2^	Redefined in TRANSCEND to align with JULIET definition
Dyspnea	Grade ≤ 1 by NCI CTCAE	Grade ≤ 1	None
Oxygen saturation	≥ 92% on room air	> 91% on room air	None
LVEF	≥ 40%	≥ 45%	Redefined in TRANSCEND to align with JULIET definition
Tumor burden	SPD (cm^2^) measured before lymphodepleting chemotherapy and at enrollment	Reported as tumor volume (mL)	No action was taken as variables were not compatible between studies (i.e., measured differently); therefore, would not be included in any subsequent analyses
Bulky disease	Single nodal mass of ≥ 10 cm by CT based on Lugano classification	NR	No action taken as variables were not compatible between studies; therefore, would not be included in any subsequent analyses
Key exclusion criteria			
Prior allo-HSCT	Allowed (not within 90 days of leukapheresis)	Not allowed	None
Active CNS lymphoma	Secondary CNS lymphoma allowed	Not allowed	None
History of other primary malignancy	Not allowed unless other primary malignancy was in remission for ≥ 2 years	Not allowed unless primary malignancy, which had been completely resected and was in complete remission for ≥ 5 years	None
Infections	Uncontrolled systemic fungal, bacterial, viral, or other infection despite appropriate antibiotics or other treatment at the time of leukapheresis or liso-cel administration	Uncontrolled acute life-threatening bacterial, viral, or fungal infection (i.e., blood culture positive ≤ 72 h before infusion)	None
Cardiovascular conditions or clinically significant cardiac disease	Within 6 months of screening/enrollment	Myocardial infarction within 6 months of screening Cardiac arrhythmia not controlled with medical management	None

*allo-HSCT* allogeneic hematopoietic stem cell transplantation, *auto-HSCT* autologous hematopoietic stem cell transplantation, *CAR* chimeric antigen receptor, *CNS* central nervous system, *CrCl* creatinine clearance, *CT* computed tomography, *DLBCL* diffuse large B-cell lymphoma, *ECOG PS* Eastern Cooperative Oncology Group performance status, *FL3B* follicular lymphoma grade 3B, *HGBCL* high-grade B-cell lymphoma, *IPD* individual patient data, *IV* intravenous, *liso-cel* lisocabtagene maraleucel, *LVEF* left ventricular ejection fraction, *NCI CTCAE* National Cancer Institute Common Terminology Criteria for Adverse Events, *NHL* non-Hodgkin lymphoma, *NOS* not otherwise specified, *NR* not reached, *PET* positron emission tomography, *PMBCL* primary mediastinal B-cell lymphoma, *R/R* relapsed or refractory, *SPD* sum of the product of perpendicular diameters, *tFL* transformed follicular lymphoma, *tiNHL* transformed indolent non-Hodgkin lymphoma, *ULN* upper limit of normal

^a^Bendamustine regimen was used if there was previous grade 4 hemorrhagic cystitis with cyclophosphamide or the patient demonstrated resistance to a previous cyclophosphamide-containing regimen. Of patients in the JULIET efficacy analysis set (n = 93), 68 received fludarabine and cyclophosphamide, 18 received bendamustine, and 8 received no lymphodepleting chemotherapy

^b^ECOG PS of 2 was allowed until Protocol Amendment 5, August 17, 2017 to align with the eligibility criteria in Abramson et al. [37]

^c^Assessed by the investigator to have had adequate bone marrow function to receive lymphodepleting chemotherapy

### Patient characteristics

Of the 17 baseline patient characteristics reported in both studies, definitions or minimum/maximum thresholds differed between the studies for nine patient characteristics. Definitions or categorizations of these patient characteristics as used in TRANSCEND were aligned to JULIET either by recategorizing or recalculating the corresponding variables from the TRANSCEND individual patient data (IPD; details presented in Table 2), thereby allowing their inclusion in analyses and reducing bias owing to differences between studies.

### Outcomes

All analyses conducted for the patient populations included those patients who were enrolled and received ≥ 1 dose of CAR T cells (ie, were infused). Outcomes of interest included efficacy (ORR, CR rate, PFS, and OS) and safety (CRS per Lee 2014 criteria, NEs per study-specified definitions [including aphasia and encephalopathy], infections, hypogammaglobulinemia, and prolonged cytopenia [defined as grade ≥ 3 cytopenias not resolved by day 29 after infusion]).

### Statistical analysis

Relevant clinical factors for matching and adjusting were identified via literature search, which was reviewed by a panel of external clinical experts. A ranked list of clinical prognostic factors and treatment-effect modifiers was derived per outcome by evaluating the strength of association between each clinical factor to each efficacy outcome endpoint (i.e., data-driven rank) using classification-based random forest models for binary outcomes (CR rate and ORR) and survival-based random forest models for time-to-event outcomes (OS and PFS) [18–20]. Data-driven ranks were then reviewed by the panel of experts and a final evidence-informed ranked list of factors was determined for each outcome by consolidating expert clinical opinion (Additional file 1: Table S1).

For a given set of ranked clinical prognostic factors and treatment-effect modifiers, separate MAICs were conducted sequentially, adjusting for one additional variable at a time, in order of ranked importance. After fitting each model, the performance and suitability of each MAIC model was assessed based on the following criteria: effective sample size (ESS; a proxy for sample size when patients are weighted, which is required to achieve a given level of precision), distribution of patient weights (wherein the goal is to avoid extreme patient weights), summary statistics (assessment of balance between study populations), and assumption of proportional hazards for OS and PFS. Balance was assessed using the absolute value of the standardized mean difference (SMD) for each covariate, a standard diagnostic for propensity score-based methods that enables comparability across factors and analyses [21]. Primary analyses were selected to strike a balance between these criteria (e.g., by retaining ESS and mitigating extreme patient weights, while adjusting for the most important factors), whereas sensitivity analyses prioritized adjustment of more factors over ESS.

After completing the matching phase of the MAIC, the remaining patients from TRANSCEND were weighted using a method-of-moments propensity score algorithm. Method-of-moments was chosen because only summary level data were available from JULIET and this method would guarantee an exact balancing of clinical factors of interest [22]. Generalized linear models for binary outcomes (i.e., ORR, CR rate, and safety outcomes) were used to estimate odds ratios (OR) and Cox proportional hazards models for time-to-event outcomes (i.e., OS and PFS) were used to estimate hazard ratios (HR).

All analyses were conducted using R Project for Statistical Computing, version 3.6.1 (R Core Team, Vienna, Austria; https://www.r-project.org/).

## Results

### Clinical factors before and after matching and adjusting

For each efficacy outcome, comparisons of clinical factors at baseline were conducted for TRANSCEND versus JULIET naively, without matching or adjusting infused patients from TRANSCEND. This exercise showed that few factors were similar (i.e., SMD < 0.1) between TRANSCEND and JULIET (Table 3). Notable differences (i.e., SMD ≥ 0.1) were observed for age, ECOG PS score, active secondary CNS lymphoma, disease histology, cell of origin, double or triple hit, prior allo-HSCT and auto-HSCT, bridging therapy, number of prior lines of therapy, R/R to last therapy, pre-lymphodepletion creatinine clearance, left ventricular ejection fraction at screening, and pre-leukapheresis absolute lymphocyte count. The matching phase of the MAIC involved removing TRANSCEND patients with primary mediastinal B-cell lymphoma or follicular lymphoma grade 3B disease histology (efficacy outcomes only), an ECOG PS of 2 at screening, secondary CNS lymphoma, or prior allo-HSCT. Bridging therapy was not matched in this analysis, as both trial protocols permitted the use of bridging therapy per investigator’s discretion. In both primary and sensitivity analyses, matching and adjusting patients from the TRANSCEND to the JULIET population produced substantial improvements in the balance of clinical factors between studies. For example, in the primary analysis of OS, the proportion of ranked clinical factors achieving SMD < 0.1 increased from the naive rate of 17.6% to 41.2%, which further improved in the sensitivity analysis to 88.2%. Factors with SMD < 0.1 after matching and adjusting in the primary analysis included International Prognostic Index score, ECOG PS, active secondary CNS lymphoma, disease histology, prior allo- and auto-HSCT, and R/R to last therapy. Similar improvements in balance were observed in the primary analyses conducted for ORR, CR rate, and PFS (Additional file 1: Tables S2–S4).

**Table 3:** Comparison of clinical factors before and after MAIC of OS in TRANSCEND and JULIET

Clinical factor	JULIET (tisagenlecleucel) safety set/full analysis set [8]	TRANSCEND (liso-cel) DLBCL efficacy set [6]					
		Before MAIC (naive)		After MAIC (primary)		After MAIC (sensitivity)	
ESS, N	111	256		180		24.8	

		Unadjusted	SMD	Adjusted	SMD	Adjusted	SMD
Mean (SD) age, years	53.9 (12.9)	60.3 (13.3)	0.483	61.3 (11.8)	0.605	53.9 (13.1)	0.000
Male sex, %	61.3	66.0	0.098	66.5	0.108	61.3	0.000
IPI score, categorized per JULIET categorization, %							
0‒1	27.9	24.6	0.070	28.7	0.020	27.9	0.000
2‒5	72.1	74.6	–	70.9	–	72.1	–
Missing	0	0.8	–	0.3	–	0	–
ECOG PS score at screening, %							
0	55.0	40.6	0.331	55.0	0.000	55.0	0.000
1	45.0	57.8	–	45.0	–	45.0	–
2	0	1.6	–	0	–	0	–
Disease stage, %							
I or II	24.3	27.0	0.066	30.5	0.141	24.3	0.000
III or IV	75.7	72.3	–	69.2	–	75.7	–
Missing	0	0.8	–	0.3	–	0	–
Secondary CNS lymphoma at time of treatment, %							
No	100	97.7	0.219	100	0.000	100	0.000
Yes	0	2.3	–	0	–	0	–
Disease histology, categorized per JULIET categorization, %							
DLBCL	81.1	71.1	0.397	81.1	0.000	81.1	0.000
DLBCL tFL	18.9	22.3	–	18.9	–	18.9	–
PMBCL	0	5.5	–	0	–	0	–
FL3B	0	1.2	–	0	–	0	–
Cell of origin, %							
GCB	56.8	44.1	0.630	45.2	0.604	56.8	0.000
ABC	40.5	28.1	–	33.2	–	40.5	–
Unknown	2.7	21.1	–	21.6	–	2.7	–
Missing	0	6.6	–	0	–	0.0	–
Double or triple hit, %							
Unknown	36.9	29.3	0.202	28.6	0.219	37.0	0.000
No	45.9	55.9	–	56.6	–	45.9	–
Yes	17.1	14.8	–	14.8	–	17.1	–
Prior allo-HSCT, %							
No	100	97.3	0.237	100	0.000	100	0.000
Yes	0	2.7	–	0	–	0	–
Prior auto-HSCT, %							
No	51.4	66.8	0.317	51.4	0.000	51.4	0.000
Yes	48.6	33.2	–	48.6	–	48.6	–
Bridging therapy, %							
No	8.1	41.4	0.837	47.4	0.977	52.4	1.101
Yes	91.9	58.6	–	52.6	–	47.6	–
Number of prior lines of therapy, per JULIET definition, %							
1	4.5	0.4	0.859	0.6	0.894	0	0.315
2	44.1	19.5	–	16.9	–	48.7	–
3	30.6	26.6	–	28.0	–	30.6	–
4‒6	20.7	43.4	–	46.1	–	20.7	–
≥ 7	0	9.8	–	8.4	–	0	–
Missing	0	0.4	–	0	–	0	–
R/R to last therapy, per JULIET definition, %							
Refractory	55.0	63.7	0.182	55.0	0.000	55.0	0.000
Relapsed	45.0	35.9	–	45.0	–	45.0	–
Missing	0	0.4	–	0	–	0	–
CrCl pre-lymphodepletion, per JULIET criteria, %							
< 60 mL/min	0	19.1	0.688	22.3	0.757	0	0.000
≥ 60 mL/min	100	80.9	–	77.7	–	100.0	–
LVEF at screening, per JULIET criteria, %							
< 45%	0	1.6	0.178	1.4	0.170	0	0.000
≥ 45%	100	98.4	–	98.6	–	100.0	–
ALC pre-leukapheresis, per JULIET criteria, %							
< 0.3	0	10.5	0.501	10.1	0.491	0	0.000
≥ 0.3	100	84.0	–	83.6	–	100.0	–
Missing	0	5.5	–	6.3	–	0	–
Statistics, %							
Factors with SMD < 0.2,	–	29.4	–	58.8	–	88.2	–
Factors with SMD < 0.1,	–	17.6	–	41.2	–	88.2	–

*ABC* activated B cell,* ALC* absolute lymphocyte count,* allo-HSCT* allogeneic hematopoietic stem cell transplantation,* auto-HSCT* autologous hematopoietic stem cell transplantation,* CNS* central nervous system,* CrCl* creatinine clearance,* DLBCL* diffuse large B-cell lymphoma,* ECOG PS* Eastern Cooperative Oncology Group performance status,* ESS* effective sample size,* FL3B* follicular lymphoma grade 3B,* GCB* germinal center B cell,* HSCT* hematopoietic stem cell transplantation,* IPI* International Prognostic Index,* liso-cel* lisocabtagene maraleucel,* LVEF* left ventricular ejection fraction,* MAIC* matching-adjusted indirect comparison,* OS* overall survival,* PMBCL* primary mediastinal B-cell lymphoma,* R/R* relapsed or refractory,* SD* standard deviation,* SMD* standardized mean difference,* tFL* transformed follicular lymphoma

There were six clinical prognostic factors and treatment-effect modifiers used in the primary efficacy analyses. While the adjustment factors differed for each efficacy outcome, the matching criteria of disease histology, ECOG PS, secondary CNS lymphoma, and prior allo-HSCT were consistently used across the primary efficacy analyses. All available clinical factors except bridging therapy were adjusted for in the sensitivity analyses (Additional file 1: Table S1). There were four clinical factors adjusted for in the primary safety analysis; three clinical factors (secondary CNS lymphoma, ECOG PS, and prior allo-HSCT) were related to trial eligibility criteria and were used to match the TRANSCEND and JULIET populations. One additional clinical factor (number of prior therapies) was then adjusted to minimize differences between studies in the remaining patients according to the final rank-order for all safety outcomes (Additional file 1: Table S5).

### Efficacy analyses

Overall, the results of the MAIC showed a statistically significant greater odds of response for liso-cel than for tisagenlecleucel. Naive ORRs were higher for liso-cel (72.7% [n = 256]) than for tisagenlecleucel (51.6% [n = 93]) (Table 4). This corresponded to significantly greater odds of overall response for liso-cel than for tisagenlecleucel (OR = 2.49, 95% confidence interval [CI]: 1.52‒4.07; *P* < 0.001). In the primary analysis that matched and adjusted for six factors, liso-cel had an ORR of 74.7% (ESS = 164). The odds of overall response were significantly greater for liso-cel than for tisagenlecleucel (OR = 2.78, 95% CI: 1.63‒4.74; *P* < 0.001). Similarly, in the sensitivity analysis that matched and adjusted for all available clinical factors except for bridging therapy, liso-cel had an ORR of 80.8% (ESS = 37.3). The odds of overall response were, again, significantly greater for liso-cel than for tisagenlecleucel (OR = 3.95; 95% CI: 1.64‒9.51; *P* = 0.002).

**Table 4:** ORR and CR rate MAIC results for the comparison of liso-cel to tisagenlecleucel, infused patients

	JULIET (tisagenlecleucel) efficacy analysis set [8]		TRANSCEND (liso-cel) DLBCL efficacy set [6]		Liso-cel vs tisagenlecleucel	
	n	%	n or ESS	%	OR (95% CI)	*P*-value
ORR analyses						
Naive	93	51.6	256	72.7	2.49 (1.52‒4.07)	< 0.001
Primary			164	74.7	2.78 (1.63‒4.74)	< 0.001
Sensitivity			37.3	80.8	3.95 (1.64‒9.51)	0.002
CR rate analyses						
Naive	93	39.8	256	53.1	1.71 (1.06‒2.78)	0.029
Primary			200.1	57.0	2.01 (1.22‒3.30)	0.006
Sensitivity			37.3	60.6	2.33 (1.06‒5.10)	0.034

*CR* complete response, *DLBCL* diffuse large B-cell lymphoma, *ESS* effective sample size, *liso-cel* lisocabtagene maraleucel, *MAIC* matching-adjusted indirect comparions, *OR* odds ratio, *ORR* objective response rate

Naive CR rates were higher for liso-cel (53.1% [n = 256]) than for tisagenlecleucel (39.8% [n = 93]; Table 4). This corresponded to significantly greater odds of CR for liso-cel than for tisagenlecleucel (OR = 1.71, 95% CI: 1.06‒2.78; *P* = 0.029). In the primary analysis (six factors), liso-cel was associated with a CR rate of 57.0% (ESS = 200.1). The odds of CR were significantly greater for liso-cel than for tisagenlecleucel (OR = 2.01, 95% CI: 1.22‒3.30; *P* = 0.006). In the sensitivity analysis (all available clinical factors except for bridging therapy), liso-cel had a CR rate of 60.6% (ESS = 37.3). The odds of CR were also significantly greater for liso-cel than for tisagenlecleucel (OR = 2.33; 95% CI: 1.06‒5.10; *P* = 0.034).

**Fig. 1:**
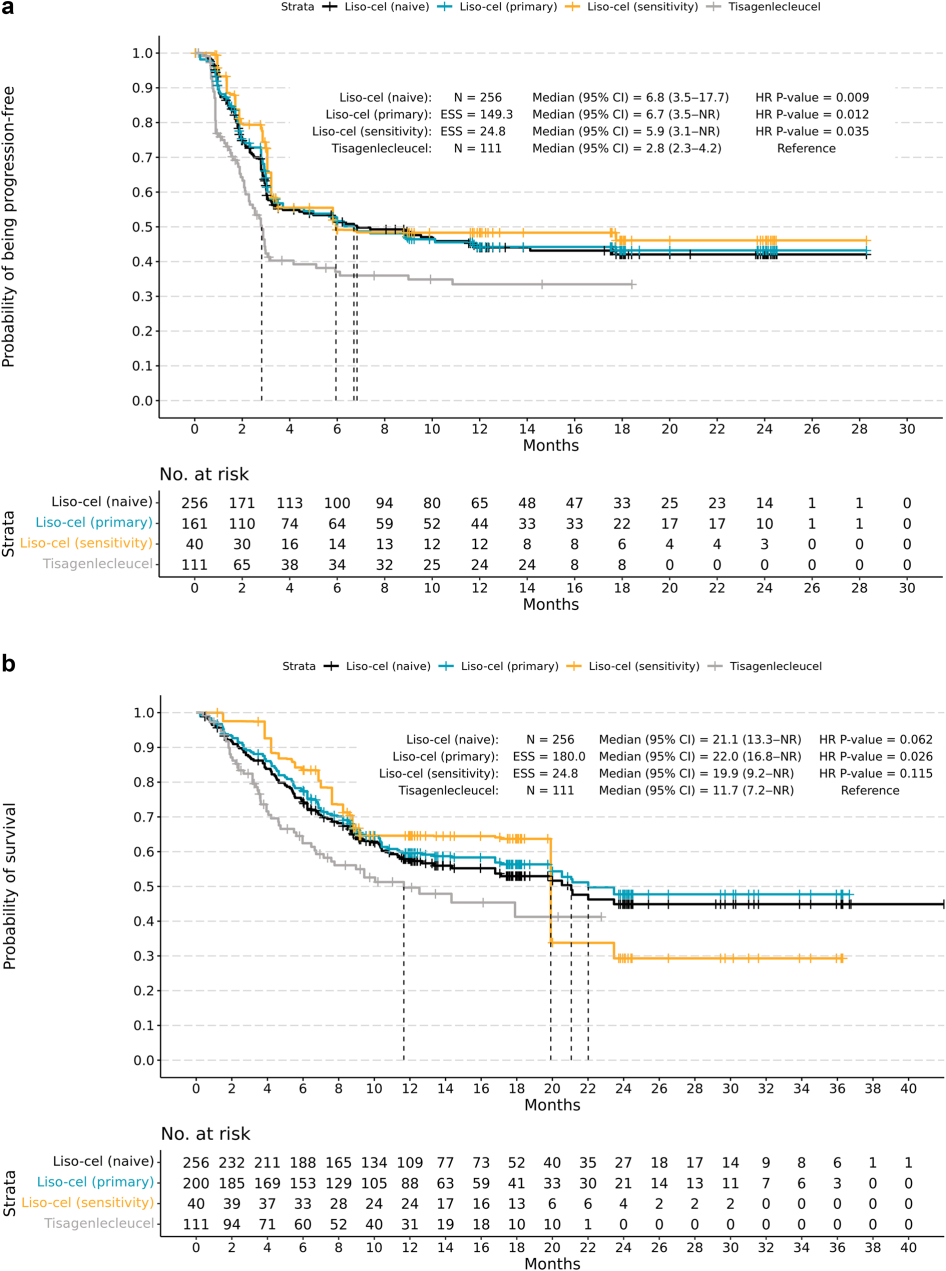
Kaplan–Meier curves for PFS (**a**) and OS (**b**) in infused patients, matched-adjusted comparison (primary analysis). *CI* confidence interval, *ESS* effective sample size, *liso-cel* lisocabtagene maraleucel, *NR* not reached, *OS* overall survival, *PFS* progression-free survival

In naive comparisons, liso-cel had a longer median PFS (6.8 months; 95% CI: 3.5‒17.7; N = 256) than tisagenlecleucel (2.8 months; 95% CI: 2.3‒4.2; N = 111). This corresponded to a significantly lower rate of disease progression for liso-cel than for tisagenlecleucel (HR = 0.67, 95% CI: 0.49‒0.91; *P* = 0.009; Table 5). In the primary analysis (six factors), liso-cel had a median PFS of 6.7 months (95% CI: 3.5‒not reached [NR]; ESS = 149.3). The rate of disease progression was significantly lower for liso-cel than for tisagenlecleucel (HR = 0.65, 95% CI: 0.47‒0.91; *P* = 0.012; Fig. 1a). In the sensitivity analysis (all available clinical factors except for bridging therapy), the median PFS for liso-cel was 5.9 months (95% CI: 3.1‒NR; ESS = 24.8). Similar to the primary analysis, the rate of disease progression was significantly lower for liso-cel than for tisagenlecleucel (HR = 0.55; 95% CI: 0.32‒0.96; *P* = 0.035; Table 5).

**Table 5:** PFS and OS MAIC results for the comparison of liso-cel to tisagenlecleucel, infused patients

	JULIET (tisagenlecleucel) efficacy analysis set [8]		TRANSCEND (liso-cel) DLBCL efficacy set [6]		Liso-cel vs tisagenlecleucel	
	N	Median, months (95% CI)	n or ESS	Median, months (95% CI)^a^	HR (95% CI)	*P-*value
PFS analyses						
Naive	111	2.8 (2.3‒4.2)^b^	256	6.8 (3.5‒17.7)	0.67 (0.49‒0.91)	0.009
Primary			149.3	6.7 (3.5‒NR)	0.65 (0.47‒0.91)	0.012
Sensitivity			24.8	5.9 (3.1‒NR)	0.55 (0.32‒0.96)	0.035
OS analyses						
Naive	111	11.7 (7.2‒NR)^b^	256	21.1 (3.3‒NR)	0.73 (0.52‒1.02)	0.062
Primary			180	22.0 (16.8‒NR)	0.67 (0.47‒0.95)	0.026
Sensitivity			24.8	19.9 (9.2‒NR)	0.68 (0.42‒1.10)	0.115

*CI* confidence interval, *DLBCL* diffuse large B-cell lymphoma, *ESS* effective sample size, *HR* hazard ratio, *IPD* individual patient data, *liso-cel* lisocabtagene maraleucel, *MAIC* matching-adjusted indirect comparison, *NR* not reached, *OS* overall survival, *PFS* progression-free survival

^a^CIs for the medians were estimated using cumulative hazard function

^b^The median was obtained from pseudo-IPD based on a digitized Kaplan–Meier curve

In naive comparisons, liso-cel had a longer median OS (21.1 months; 95% CI: 13.3‒NR; N = 256) than tisagenlecleucel (11.7 months; 95% CI: 7.2‒NR; N = 111) but the mortality rate was not significantly different between treatments (HR = 0.73, 95% CI: 0.52‒1.02; *P* = 0.062; Table 5). In the primary analysis (six factors), liso-cel had a median OS of 22.0 months (95% CI: 16.8‒NR; ESS = 180.0). For this comparison, the mortality rate was significantly lower for liso-cel than for tisagenlecleucel (HR = 0.67, 95% CI: 0.47‒0.95; *P* = 0.026; Fig. 1b). In the sensitivity analysis (all available clinical factors except for bridging therapy), median OS for liso-cel was 19.9 months (95% CI: 9.2‒NR; ESS = 51.0). For this comparison, the mortality rate was not significantly different between liso-cel and tisagenlecleucel (HR = 0.68, 95% CI: 0.42‒1.10; *P* = 0.115; Table 5).

### Safety analyses

Safety analyses were conducted for the infused patient populations (TRANSCEND, N = 269; JULIET, N = 111). After matching and adjusting for four factors, the ORs for most safety endpoints were similar for both treatments or were lower for liso-cel (ESS = 122.9) than for tisagenlecleucel. Specifically, liso-cel had statistically significant lower odds after MAIC of all-grade and grade ≥ 3 CRS and grade ≥ 3 prolonged cytopenia (Table 6).

**Table 6:** MAIC results for safety outcomes in the comparison of liso-cel to tisagenlecleucel

Safety outcomes	Grades	JULIET (tisagenlecleucel) efficacy analysis set [8]	TRANSCEND (liso-cel) DLBCL efficacy set [6]		Liso-cel vs tisagenlecleucel, OR (95% CI)			
		Reported rates, %	Naive, %	MAIC, %	Naive	*P-*value	MAIC	*P-*value
		(N = 111)	(N = 269)	(ESS = 122.9)				
CRS, Lee 2014 criteria	All grades	56.8	42.0	41.1	0.55 (0.35‒0.86)	0.009	0.53 (0.32‒0.89)	0.016
	Grade ≥ 3	17.1	2.2	2.0	0.11 (0.04‒0.29)	< 0.001	0.10 (0.03‒0.31)	< 0.001
NE, per study-specific definition	All grades	21	29.7	21.0	1.59 (0.94‒2.70)	0.085	1.36 (0.76‒2.44)	0.306
	Grade ≥ 3	12	10.0	9.7	0.82 (0.41‒1.65)	0.576	0.79 (0.36‒1.73)	0.551
Encephalopathy, per study-specific definition^a^	All grades	6	6.3	6.5	1.06 (0.42‒2.67)	0.907	1.09 (0.38‒3.12)	0.867
Aphasia, per study-specific definition^a^	All grades	3	8.2	6.4	2.88 (0.89‒9.33)	0.078	2.21 (0.64‒7.61)	0.209
Infections, any pathogens, per infections and infestations SOC	Grade ≥ 3	19.8	12.3	12.1	0.57 (0.31‒1.02)	0.060	0.56 (0.28‒1.10)	0.090
Hypogammaglobulinemia^a^, grouped term	All grades	14^b^	13.8	10.0	0.98 (0.52‒1.85)	0.949	0.68 (0.33‒1.43)	0.313
Prolonged cytopenia, laboratory assessment	Grade ≥ 3	52.8^c^ (n = 106)	37.2	32.8	0.53 (0.34‒0.83)	0.006	0.44 (0.26‒0.73)	0.002

*CRS* cytokine release syndrome, *DLBCL* diffuse large B-cell lymphoma, *ESS* effective sample size, *liso-cel* lisocabtagene maraleucel, *MAIC* matching-adjusted indirect comparison, *NE* neurological event, *OR* odds ratio, *SOC* System Organ Class, *TEAE* treatment-emergent adverse event

^a^Represents TEAE as assessed by investigators

^b^Reporting time was not specified

^c^Prolonged cytopenia by laboratory assessment was reported for n = 106 (data cutoff: September 6, 2017; Kymriah [Summary Basis for Regulatory Action]) [35]. Prolonged cytopenia per investigator assessment was reported for N = 111 in Schuster et al. [8] but could not be used owing to differences in assessment approach

The naive rate of CRS per Lee 2014 criteria was lower for liso-cel than for tisagenlecleucel (Table 6) and corresponded to significantly lower odds of all-grade (OR = 0.55, 95% CI: 0.35‒0.86; *P* = 0.009) and grade ≥ 3 (OR = 0.11, 95% CI: 0.04‒0.29; *P* < 0.001) CRS events for liso-cel. After matching and adjusting (four factors), there were statistically significant lower odds of all-grade (OR = 0.53, 95% CI: 0.32‒0.89; *P* = 0.016) and grade ≥ 3 (OR = 0.10, 95% CI: 0.03‒0.31; *P* < 0.001) CRS events for liso-cel than for tisagenlecleucel.

There were no statistically significant differences in study-specific NE rates for liso-cel compared with tisagenlecleucel. The naive rate of NEs was higher for liso-cel than for tisagenlecleucel (Table 6) and corresponded to numerically greater odds of all-grade NEs for liso-cel (OR = 1.59, 95% CI: 0.94‒2.70; *P* = 0.085). The inverse was true for grade ≥ 3 NEs, for which naive rates were lower for liso-cel than for tisagenlecleucel and corresponded to numerically lower odds of grade 3 events (OR = 0.82, 95% CI: 0.41‒1.65; *P* = 0.576). After matching and adjusting, the odds of all-grade NEs were numerically greater for liso-cel (OR = 1.36, 95% CI: 0.76‒2.44; *P* = 0.306) but grade ≥ 3 NEs were numerically lower for liso-cel (OR = 0.79, 95% CI: 0.36‒1.73; *P* = 0.551).

The naive rates of study-specific NEs of all-grade encephalopathy events were similar for liso-cel and tisagenlecleucel (OR = 1.06, 95% CI: 0.42‒2.67; *P* = 0.907; Table 6). After matching and adjusting, there were no statistically significant differences in all-grade encephalopathy events. The naive rates of study-specific NEs of all-grade aphasia were numerically higher for liso-cel (OR = 2.88, 95% CI: 0.89‒9.33; *P* = 0.078). After matching and adjusting, there were no statistically significant differences in all-grade aphasia events.

Rates of laboratory-confirmed grade ≥ 3 prolonged cytopenia were significantly lower for liso-cel for both the naive (OR = 0.53, 95% CI: 0.34‒0.83; *P* = 0.006) and matching-adjusted (OR = 0.44, 95% CI: 0.26‒0.73; *P* = 0.002) data sets. There were no statistically significant differences in infection or hypogammaglobulinemia rates between liso-cel and tisagenlecleucel.

## Discussion

Liso-cel had favorable efficacy and a comparable or better safety profile relative to tisagenlecleucel after matching and adjusting for important clinical prognostic factors and treatment-effect modifiers in this MAIC. The MAIC approach is a form of population adjustment designed to mitigate between-study differences in eligibility criteria, adjust for between-study differences in baseline characteristics, reconcile differences in varying definitions, and reduce sensitivity to effect measures. An assessment identified 17 clinical factors reported in both TRANSCEND and JULIET that were available for adjustment. The primary efficacy analysis that matched on and adjusted for six clinical factors showed that the odds of response were significantly greater, while the odds of disease progression and mortality were significantly lower for liso-cel than tisagenlecleucel. To assess the robustness of the primary efficacy analysis, sensitivity analyses were conducted by matching and adjusting for all available clinical factors except for bridging therapy, at the expense of ESS. Sensitivity analyses supported the primary findings, except for OS, for which there was no longer a statistically significant greater OS for liso-cel. However, because the sensitivity analyses adjusted for more factors, the corresponding estimates were based on a lower ESS, which produced greater uncertainty in statistical estimates (i.e., wider CIs). Furthermore, a large drop in the sensitivity analysis Kaplan–Meier curve for OS was estimated at around 20 months because of loss to follow-up (i.e., censoring), accentuating a large patient weight in the remaining risk set.

After matching and adjusting for four clinical factors, the ORs for most safety endpoints were similar for both treatments or were lower for liso-cel than for tisagenlecleucel. Importantly, liso-cel had statistically significant lower odds after MAIC of all-grade and grade ≥ 3 CRS and grade ≥ 3 prolonged cytopenia.

Two MAIC analyses assessing the relative efficacy and safety between axi-cel (ZUMA-1) and tisagenlecleucel (JULIET) have been performed. A recently published MAIC by Oluwole et al. matched and adjusted the ZUMA-1 population to JULIET [23]. The authors found that, after adjusting for differences in patient characteristics between studies, axi-cel was associated with a higher ORR and CR rate than tisagenlecleucel among patients who underwent infusion, and OS comparisons favored axi-cel. They also found a higher rate of grades 1–2 CRS in ZUMA-1 compared with JULIET, though similar rates of grade ≥ 3 CRS and study-specific NEs. However, they noted significant limitations that could have led to bias since definitions of relapsed disease differed and they could not account for the impact bridging chemotherapy had on relative outcomes. In contrast, Zhang et al. matched and adjusted the JULIET population to ZUMA-1 [24]. The authors concluded that differences between the JULIET and ZUMA-1 patient populations were substantial, rendering estimates of relative treatment effects (via MAIC or other adjusted indirect treatment comparison methods) unreliable, due to small ESSs, after aligning patient population data sets. For example, matching on bridging therapy alone (0% in ZUMA-1 and > 90% in JULIET) would have resulted in < 10% of patients remaining in IPD from JULIET. Furthermore, the authors discussed sources of bias that could not be accounted for in statistical analyses, such as manufacturing times in the enrollment process, which could undermine the accuracy of indirect treatment comparison estimates.

A recent MAIC analysis comparing efficacy-evaluable patients in JULIET (N = 115; data cutoff February 2020) to TRANSCEND (N = 256; data cutoff August 2019) was conducted to evaluate the comparative efficacy of tisagenlecleucel versus liso-cel, and found no evidence of differences in ORR, CR rate, OS, and PFS between the two CAR T-cell therapies [25, 26]. Several of the following analytical approaches employed by the authors are worth noting: (1) 8 patients who did not receive lymphodepleting chemotherapy and 1 patient with DLBCL misclassification were first removed from the JULIET dataset before analysis (n = 106); (2) TRANSCEND enrolled a broader patient population (e.g., primary mediastinal B-cell lymphoma and follicular lymphoma grade 3B subtypes, ECOG PS of 2, secondary CNS lymphoma, prior allo-HSCT, impaired renal function, no prespecified threshold for blood counts) that could not be emulated using patients enrolled in JULIET; (3) proportion of patients who did not receive bridging therapy in JULIET (n = 11 of 106) was up-weighted from 10.4% to 42.4% to match that from TRANSCEND (n = 106 of 256). MAIC resulted in an ESS of 29 compared with an initial sample of 106 patients in the JULIET study. The low ESS may be because of the initial small sample size of JULIET and/or matching to the proportion of patients receiving bridging therapy in TRANSCEND. A small ESS is an indication that the patient weights are highly variable owing to a lack of population overlap, and that the estimate may be unstable. The distribution of weights themselves should also be examined directly alongside pre- and post-MAIC balance in baseline characteristics (e.g., via SMDs) to diagnose population overlap and to highlight any overly influential individuals. Furthermore, there is an additional challenge to directly compare patients who received bridging therapy in the two studies, since the manufacturing times differed for the two CAR T-cell therapies (median time from enrollment to infusion in JULIET: 54 days [90% of patients received infusions between 30 and 92 days after enrollment] vs median time from leukapheresis to infusion in TRANSCEND: 37 days [range, 27‒224 days]). As bridging therapy was administered at the discretion of the investigator, the shorter time to CAR T-cell availability for liso-cel may have resulted in bridging therapy being administered preferentially to patients with more aggressive or rapidly progressing disease, whereas the longer time to CAR T-cell availability for tisagenlecleucel may have resulted in administering bridging therapy to a broader group of patients, as 90% of patients received bridging therapy in JULIET versus 59% of patients in TRANSCEND. Taken together, this suggests that the author's main findings are unlikely applicable to the intended target population represented by TRANSCEND. Given these limitations, the study design was likely insufficient for detecting clinically relevant effect sizes for the intended comparison. This highlights the importance of compatibility assessment between trials as a first step for an MAIC. As we have shown in our analysis, the broader patient population of TRANSCEND is better suited for matching and adjusting to the JULIET patient population, resulting in a higher degree of alignment for comparisons.

Our MAIC analysis has several notable strengths. Multiple JULIET data sources were evaluated to identify the most compatible cohorts to those available in the TRANSCEND pivotal trial for each outcome. The analysis employed a rigorous, multifaceted process to identify and rank-order clinically relevant factors. Clinical experts rank-ordered factors from most to least important to include in our models, which, when paired with data-driven rankings, resulted in the final list of evidence-informed ranking of factors. Accounting for these challenges to matching and adjustment, the ESS remained robust enough to allow for clinically relevant conclusions about the comparison of these two CAR T-cell therapies. However, there were several limitations we should note. Absence of a common comparator in TRANSCEND and JULIET meant that only an unanchored MAIC could be performed. Given the degree of imbalance between the two studies, it was not feasible to match and adjust on all identified factors without losing substantial ESS. Though rank-ordering the factors helped ensure the most important factors were prioritized for inclusion in the model, only a subset of those identified could be included in the primary analyses. Enrollment and manufacturing times differed between studies; the impact of differences in manufacturing process and time could not be fully accounted for in the analysis, which has been posited as a potentially significant bias factor [24]. The inability to control for the factors of bulky disease and tumor burden may have impacted the overall results. Finally, though sensitivity analyses involving additional clinical factors offered alternative estimated relative treatment effects, they often relied on reduced ESS. This manifested in less-reliable Kaplan–Meier curve estimates at longer follow-up times, where the number-at-risk set is small and estimation was predominantly based upon a few patients. Despite these limitations, it is encouraging that the primary and sensitivity analyses were similar, indicating a statistically significant efficacy advantage for liso-cel compared with tisagenlecleucel, with the exception of the sensitivity analysis for OS.

While the liso-cel and tisagenlecleucel CAR constructs both contain a 4-1BB costimulatory domain, there are differences in the CAR T-cell manufacturing process and composition of the two products. The liso-cel manufacturing process purifies T cells from the leukapheresis to minimize tumor cell residuals and includes T-cell–specific activation for a consistent reduction of non–T-cell impurities. CD8^+^ and CD4^+^ cells are positively selected from fresh leukapheresis, and each population is separately activated, transduced, and expanded. Liso-cel is a defined composition product administered as a sequential infusion of separate CD8^+^ and CD4^+^ components at equal target doses. Preclinical studies have show that CD4^+^ cells affect CD8^+^ effector T-cell expansion, memory formation, trafficking, and cytolytic effector T-cell function [27–29], indicating that at least some CD8^+^ function may be optimized by controlling the dose of CD8^+^ and CD4^+^ cell components [30]. In animal models, a 1:1 ratio of CD8^+^:CD4^+^ CAR T cells showed improved expansion and activity over treatment with either T-cell component alone [31]. The tisagenlecleucel manufacturing process begins with a frozen leukapheresis sample, after which the vector is introduced into T cells selected from thawed peripheral blood mononuclear cells using CD3/CD28-coated magnetic beads. Unlike the liso-cel manufacturing process, tisagenlecleucel manufacturing does not select for the T-cell subpopulations [32], leading to heterogeneity of the CD8^+^:CD4^+^ ratio in the final product. The cellular composition and final cell number vary between individual patient batches. This heterogeneity may contribute to differences in efficacy and safety profiles of the different CAR T-cell products.

## Conclusions

In summary, an unanchored MAIC leveraging IPD from TRANSCEND and summary level data from JULIET was used to derive indirect comparisons while accounting for between-study differences in eligibility criteria and baseline characteristics. Overall, after matching and adjusting for important clinical prognostic factors and treatment-effect modifiers, liso-cel had favorable efficacy and a comparable or better safety profile relative to tisagenlecleucel. This analysis, which was bound by the context and limitations of the single-arm studies, does not replace a head-to-head, randomized controlled study, and these results should be further validated in a real-world clinical setting.

## Supplementary Information

**Additional file 1: Table S1**. Clinical factors included for primary and sensitivity analyses comparing liso-cel with tisagenlecleucel. **Table S2**. Comparison of clinical factors before and after MAIC for primary and sensitivity analyses of PFS in TRANSCEND and JULIET. **Table S3**. Comparison of clinical factors before and after MAIC for primary and sensitivity analyses of CR in TRANSCEND and JULIET. **Table S4**. Comparison of clinical factors before and after MAIC for primary and sensitivity analyses of ORR in TRANSCEND and JULIET. **Table S5.** Comparison of clinical factors before and after MAIC for safety analysis in TRANSCEND and JULIET.

## Data Availability

Bristol Myers Squibb policy on data sharing may be found at https://www.bms.com/researchers-and-partners/independent-research/data-sharing-request-process.html.

## Abbreviations

ABC: Activated B cell
AE: Adverse event
ALC: Absolute lymphocyte count
allo-HSCT: Allogeneic hematopoietic stem cell transplantation
auto-HSCT: Autologous hematopoietic stem cell transplantation
axi-cel: Axicabtagene ciloleucel
CAR: Chimeric antigen receptor
CI: Confidence interval
CNS: Central nervous system
CR: Complete response
CrCl: Creatinine clearance
CRS: Cytokine release syndrome
CT: Computed tomography
DLBCL: Diffuse large B-cell lymphoma
ECOG PS: Eastern Cooperative Oncology Group performance status
EMA: European Medicines Agency
EOS: End of study
ESS: Effective sample size
FDA: Food and Drug Administration
FL3B: Follicular lymphoma grade 3B
GCB: Germinal center B cell
HGBCL: High-grade B-cell lymphoma
HR: Hazard ratio
IPD: Individual patient data
IPI: International Prognostic Index
IRC: Independent review committee
IV: Intravenous
LBCL: Large B-cell lymphoma
liso-cel: Lisocabtagene maraleucel
LVEF: Left ventricular ejection fraction
MAIC: Matching-adjusted indirect comparison
NCI CTCAE: National Cancer Institute Common Terminology Criteria for Adverse Events
NE: Neurological event
NHL: Non-Hodgkin lymphoma
NOS: Not otherwise specified
NR: Not reached
OR: Odds ratio
ORR: Objective response rate
OS: Overall survival
PET: Positron emission tomography
PFS: Progression-free survival
PMBCL: Primary mediastinal B-cell lymphoma
R/R: Relapsed or refractory
SD: Standard deviation
SMD: Standardized mean difference
SOC: System Organ Class
SPD: Sum of the product of perpendicular diameters
TEAE: Treatment-emergent adverse event
tFL: Transformed follicular lymphoma
tiNHL: Transformed from indolent non-Hodgkin lymphoma
ULN: Upper limit of normal
