# Supplementary material for: Matching-adjusted indirect treatment comparison of chimeric antigen receptor T-cell therapies for third-line or later treatment of relapsed or refractory large B-cell lymphoma: lisocabtagene maraleucel versus tisagenlecleucel
Source: Exp Hematol Oncol. 2022 Mar 25;11:17. doi: 10.1186/s40164-022-00268-z (PMC8953336; doi:10.1186/s40164-022-00268-z)
Supplement: Supplementary file 1 — Additional file 1: Table S1. Clinical factors included for primary and sensitivity analyses comparing liso-cel with tisagenlecleucel. Table S2. Comparison of clinical factors before and after MAIC for primary and sensitivity analyses of PFS in TRANSCEND and JULIET. Table S3. Comparison of clinical factors before and after MAIC for primary and sensitivity analyses of CR in TRANSCEND and JULIET. Table S4. Comparison of clinical factors before and after MAIC for primary and sensitivity analyses of ORR in TRANSCEND and JULIET. Table S5. Comparison of clinical factors before and after MAIC for safety analysis in TRANSCEND and JULIET. [file 40164_2022_268_MOESM1_ESM.docx]

**Supplementary Appendix**

**Matching-adjusted indirect treatment comparison of chimeric antigen receptor T-cell therapies for third-line or later treatment of relapsed or refractory large B-cell lymphoma: lisocabtagene maraleucel versus tisagenlecleucel**

Guillaume Cartron, Christopher P. Fox, Fei Fei Liu, Ana Kostic, Jens Hasskarl, Daniel Li, Ashley Bonner, Yixie Zhang, David G. Maloney and John Kuruvilla

**Table of Contents**

[Results 3](#_Toc74731249)

[Table S1. Clinical factors included for primary and sensitivity analyses comparing liso-cel with tisagenlecleucel. 3](#_Toc74731250)

[Table S2. Comparison of clinical factors before and after MAIC for primary and sensitivity analyses of PFS in TRANSCEND and JULIET. 4](#_Toc74731251)

[Table S3. Comparison of clinical factors before and after MAIC for primary and sensitivity analyses of CR in TRANSCEND and JULIET. 9](#_Toc74731252)

[Table S4. Comparison of clinical factors before and after MAIC for primary and sensitivity analyses of ORR in TRANSCEND and JULIET. 13](#_Toc74731253)

[Table S5. Comparison of clinical factors before and after MAIC for safety analysis in TRANSCEND and JULIET. 17](#_Toc74731254)

Results

| Table S1 Clinical factors included for primary and sensitivity analyses comparing liso-cel with tisagenlecleucel | | | | | | | | | |
| --- | --- | --- | --- | --- | --- | --- | --- | --- | --- |
|  |  | **ORR** | | **CR rate** | | **PFS** | | **OS** | |
|  | **Clinical factors** | **Pri** | **Sens** | **Pri** | **Sens** | **Pri** | **Sens** | **Pri** | **Sens** |
| Factors matched on | Disease histology | ✓ | ✓ | ✓ | ✓ | ✓ | ✓ | ✓ | ✓ |
|  | ECOG PS | ✓ | ✓ | ✓ | ✓ | ✓ | ✓ | ✓ | ✓ |
|  | Secondary CNS lymphoma | ✓ | ✓ | ✓ | ✓ | ✓ | ✓ | ✓ | ✓ |
|  | Prior allo-HSCT | ✓ | ✓ | ✓ | ✓ | ✓ | ✓ | ✓ | ✓ |
| Factors adjusted for | Prior auto-HSCT | ✓ | ✓ | ✓ | ✓ |  | ✓ | ✓ | ✓ |
|  | R/R status to last therapy |  | ✓ | ✓ | ✓ | ✓ | ✓ | ✓ | ✓ |
|  | Number of prior therapies |  | ✓ |  | ✓ |  | ✓ |  | ✓ |
|  | Age |  |  |  |  |  | ✓ |  | ✓ |
|  | IPI score |  | ✓ |  | ✓ |  | ✓ |  | ✓ |
|  | Absolute lymphocyte count |  | ✓ |  | ✓ |  | ✓ |  | ✓ |
|  | Disease stage |  | ✓ |  | ✓ |  | ✓ |  | ✓ |
|  | CrCl | ✓ | ✓ |  | ✓ | ✓ | ✓ |  | ✓ |
|  | Sex |  |  |  |  |  | ✓ |  | ✓ |
|  | Cell of origin |  | ✓ |  | ✓ |  | ✓ |  | ✓ |
|  | LVEF |  | ✓ |  | ✓ |  | ✓ |  | ✓ |
|  | Double/triple hit |  | ✓ |  | ✓ |  | ✓ |  | ✓ |
| allo-HSCT: allogeneic hematopoietic stem cell transplantation; auto-HSCT: autologous hematopoietic stem cell transplantation; CNS: central nervous system; CR: complete response; CrCl: creatinine clearance; ECOG PS: Eastern Cooperative Oncology Group performance status; IPI: International Prognostic Index; liso-cel: lisocabtagene maraleucel; LVEF: left ventricular ejection fraction; ORR: objective response rate; OS: overall survival; PFS: progression-free survival; Pri: primary analysis; R/R: relapsed or refractory; Sens: sensitivity analysis. | | | | | | | | | |

Table S2 Comparison of clinical factors before and after MAIC of PFS in TRANSCEND and JULIET

| Clinical factor | Tisagenlecleucel (JULIET) safety set/full analysis set | Liso-cel (TRANSCEND) DLBCL efficacy set  PFS | | | | | | |  |
| --- | --- | --- | --- | --- | --- | --- | --- | --- | --- |
|  |  | Before MAIC (naive) | | After MAIC (primary) | | After MAIC (sensitivity) | | |  |
|  | N=111 | N=256 | | ESS=149.3 | | ESS=24.8 | | |  |
|  | Stat. | Stat. | SMD | Stat. | SMD | Stat. | | SMD |  |
| Age, years |  |  |  |  |  |  | |  |  |
| Mean (SD) | 53.9 (12.9) | 60.3 (13.3) | 0.483 | 59.7 (11.5) | 0.476 | 53.9 (13.1) | | 0.000 |  |
| Sex, % |  |  |  |  |  |  | |  |  |
| Male | 61.3 | 66.0 | 0.098 | 73.4 | 0.260 | 61.3 | | 0.000 |  |
| IPI score, categorized per JULIET categorization, % |  |  |  |  |  |  | |  |  |
| 0‒1 | 27.9 | 24.6 | 0.070 | 31.2 | 0.073 | 27.9 | | 0.000 |  |
| 2‒5 | 72.1 | 74.6 |  | 68.8 |  | 72.1 | |  |  |
| Missing | 0.0 | 0.8 |  | 0.0 |  | 0.0 | |  |  |
| ECOG PS at screening, % |  |  |  |  |  |  | |  |  |
| 0 | 55.0 | 40.6 | 0.331 | 55.0 | 0.000 | 55.0 | | 0.000 |  |
| 1 | 45.0 | 57.8 |  | 45.0 |  | 45.0 | |  |  |
| 2 | 0.0 | 1.6 |  | 0.0 |  | 0.0 | |  |  |
| Disease stage, % |  |  |  |  |  |  | |  |  |
| I or II | 24.3 | 27.0 | 0.066 | 30.5 | 0.140 | 24.3 | | 0.000 |  |
| III or IV | 75.7 | 72.3 |  | 69.5 |  | 75.7 | |  |  |
| Missing | 0 | 0.8 |  | 0.0 |  | 0.0 | |  |  |
| Secondary CNS lymphoma at time of treatment, % |  |  |  |  |  |  | |  |  |
| No | 100.0 | 97.7 | 0.219 | 100.0 | 0.000 | 100.0 | | 0.000 |  |
| Yes | 0.0 | 2.3 |  | 0.0 |  | 0.0 | |  |  |
| Disease histology, % |  |  |  |  |  |  | |  |  |
| DLBCL | 81.1 | 71.1 | 0.397 | 81.1 | 0.000 | 81.1 | | 0.000 |  |
| DLBCL tFL | 18.9 | 22.3 |  | 18.9 |  | 18.9 | |  |  |
| PMBCL | 0.0 | 5.5 |  | 0.0 |  | 0.0 | |  |  |
| FL3B | 0.0 | 1.2 |  | 0.0 |  | 0.0 | |  |  |
| Cell of origin, % |  |  |  |  |  |  | |  |  |
| GCB | 56.8 | 44.1 | 0.630 | 46.6 | 0.591 | 56.8 | | 0.000 |  |
| ABC | 40.5 | 28.1 |  | 32.4 |  | 40.5 | |  |  |
| Unknown | 2.7 | 21.1 |  | 21.1 |  | 2.7 | |  |  |
| Missing | 0 | 6.6 |  | 0.0 |  | 0.0 | |  |  |
| Double or triple hit, % |  |  |  |  |  |  | |  |  |
| Unknown | 36.9 | 29.3 | 0.202 | 25.4 | 0.265 | 37.0 | | 0.000 |  |
| No | 45.9 | 55.9 |  | 57.5 |  | 45.9 | |  |  |
| Yes | 17.1 | 14.8 |  | 17.0 |  | 17.1 | |  |  |
| Prior HSCT, % |  |  |  |  |  |  | |  |  |
| No | 100.0 | 97.3 | 0.237 | 100.0 | 0.000 | 100.0 | | 0.000 |  |
| Yes | 0.0 | 2.7 |  | 0.0 |  | 0.0 | |  |  |
| Prior auto-HSCT, % |  |  |  |  |  |  | |  |  |
| No | 51.4 | 66.8 | 0.317 | 65.2 | 0.282 | 51.4 | | 0.000 |  |
| Yes | 48.6 | 33.2 |  | 34.8 |  | 48.6 | |  |  |
| Bridging therapy, % |  |  |  |  |  |  | |  |  |
| No | 8.1 | 41.4 | 0.837 | 45.1 | 0.923 | 52.4 | | 1.101 |  |
| Yes | 91.9 | 58.6 |  | 54.9 |  | 47.6 | |  |  |
| Number of prior lines of systemic therapy, recalculated per JULIET definition, % |  |  |  |  |  |  | |  |  |
| 1 | 4.5 | 0.4 | 0.859 | 1.1 | 0.727 | 0.0 | | 0.315 |  |
| 2 | 44.1 | 19.5 |  | 22.6 |  | 48.7 | |  |  |
| 3 | 30.6 | 26.6 |  | 27.0 |  | 30.6 | |  |  |
| 4‒6 | 20.7 | 43.4 |  | 43.2 |  | 20.7 | |  |  |
| 7+ | 0.0 | 9.8 |  | 6.1 |  | 0.0 | |  |  |
| Missing | 0.0 | 0.4 |  | 0.0 |  | 0.0 | |  |  |
| Relapsed or refractory to last therapy, recalculated per JULIET definition, % |  |  |  |  |  |  |  | |  |
| Refractory | 55.0 | 63.7 | 0.182 | 55.0 | 0.000 | 55.0 | 0.000 | |  |
| Relapsed | 45.0 | 35.9 |  | 45.0 | 0.000 | 45.0 | 0.000 | |  |
| Missing | 0.0 | 0.4 |  | 0.0 |  | 0.0 |  | |  |
| Creatinine clearance prior to lymphodepleting chemotherapy, categorized per JULIET eligibility criteria, % |  |  |  |  |  |  |  | |  |
| <60 mL/min | 0.0 | 19.1 | 0.688 | 0.0 | 0.000 | 0.0 | 0.000 | |  |
| $\geq$60 mL/min | 100.0 | 80.9 |  | 100.0 |  | 100.0 |  | |  |
| Left ventricle ejection fraction at screening, categorized per JULIET eligibility criteria, % |  |  |  |  |  |  |  | |  |
| <45% | 0.0 | 1.6 | 0.178 | 1.8 | 0.192 | 0.0 | 0.000 | |  |
| ≥45% | 100.0 | 98.4 |  | 98.2 |  | 100.0 |  | |  |
| Pre-leukapheresis lymphocyte count (10^9^/L), categorized per JULIET eligibility criteria, % |  |  |  |  |  |  |  | |  |
| <0.3 | 0.0 | 10.5 | 0.501 | 10.1 | 0.500 | 0.0 | 0.000 | |  |
| ≥0.3 | 100.0 | 84.0 |  | 81.1 |  | 100.0 |  | |  |
| Missing | 0.0 | 5.5 |  | 8.7 |  | 0.0 |  | |  |
| Statistics |  |  |  |  |  |  |  | |  |
| % of factors with SMD <0.2 |  | 29.4 |  | 52.9 |  | 88.2 |  | |  |
| % of factors with SMD <0.1 |  | 17.6 |  | 41.2 |  | 88.2 |  | |  |
| ABC: activated B cell; allo-HSCT: allogenic hematopoietic stem cell transplantation; auto-HSCT: autologous hematopoietic stem cell transplantation; CNS: central nervous system; DLBCL: diffuse large B-cell lymphoma; ECOG PS: Eastern Cooperative Oncology Group performance status; ESS: effective sample size; FL3B: follicular lymphoma grade 3B; GCB: germinal center B cell; HSCT: hematopoietic stem cell transplantation; IPI: International Prognostic Index; MAIC: matching-adjusted indirect comparison; N: sample size; OS: overall survival; PFS: progression-free survival; PMBCL: primary mediastinal B-cell lymphoma; SD: standard deviation; SMD: standardized mean difference; tFL: transformed follicular lymphoma. | | | | | | | | | |

Table S3 Comparison of clinical factors before and after MAIC of CR rate in TRANSCEND and JULIET

| Clinical factor | Tisagenlecleucel (JULIET) efficacy analysis set | Liso-cel (TRANSCEND) DLBCL efficacy set  CR rate | | | | | |
| --- | --- | --- | --- | --- | --- | --- | --- |
|  |  | Before MAIC (naive) | | After MAIC (primary) | | After MAIC (sensitivity) | |
|  | N=93 | N=256 | | ESS=200.1 | | ESS=37.3 | |
|  | Stat. | Stat. | SMD | Stat. | SMD | Stat. | SMD |
| IPI score, categorized per JULIET categorization, % |  |  |  |  |  |  |  |
| 0‒1 | 26.9 | 24.6 | 0.048 | 26.4 | 0.009 | 26.9 | 0.000 |
| 2‒5 | 73.1 | 74.6 |  | 73.1 |  | 73.1 |  |
| Missing | 0.0 | 0.8 |  | 0.5 |  | 0.0 |  |
| ECOG PS at screening, % |  |  |  |  |  |  |  |
| 0‒1 | 100 | 98.4 | 0.178 | 100.0 | 0.000 | 100.0 | 0.000 |
| 2 | 0.0 | 1.6 |  | 0.0 |  | 0.0 |  |
| Disease stage, % |  |  |  |  |  |  |  |
| I or II | 22.6 | 27.0 | 0.106 | 29.1 | 0.153 | 22.6 | 0.000 |
| III or IV | 77.4 | 72.3 | 0.106 | 70.3 |  | 77.4 |  |
| Missing | 0.0 | 0.8 |  | 0.5 |  | 0.0 |  |
| Secondary CNS lymphoma at time of treatment, % |  |  |  |  |  |  |  |
| No | 100.0 | 97.7 | 0.219 | 100.0 | 0.000 | 100.0 | 0.000 |
| Yes | 0.0 | 2.3 |  | 0.0 |  | 0.0 |  |
| Disease histology, % |  |  |  |  |  |  |  |
| DLBCL | 80.6 | 71.1 | 0.393 | 77.0 | 0.088 | 80.6 | 0.000 |
| DLBCL tFL | 19.4 | 22.3 |  | 23.0 |  | 19.4 |  |
| PMBCL | 0.0 | 5.5 |  | 0.0 |  | 0.0 |  |
| FL3B | 0.0 | 1.2 |  | 0.0 |  | 0.0 |  |
| Cell of origin, % |  |  |  |  |  |  |  |
| GCB | 53.8 | 44.1 | 0.615 | 47.2 | 0.582 | 53.8 | 0.000 |
| ABC | 43.0 | 28.1 |  | 31.4 |  | 43.0 |  |
| Unknown | 3.2 | 21.1 |  | 21.3 |  | 3.2 |  |
| Missing | 0.0 | 6.6 |  | 0.0 |  | 0.0 |  |
| Double or triple hit, % |  |  |  |  |  |  |  |
| Unknown | 37.6 | 29.3 | 0.217 | 26.9 | 0.276 | 37.6 | 0.000 |
| No | 45.2 | 55.9 |  | 58.6 |  | 45.2 |  |
| Yes | 17.2 | 14.8 |  | 14.6 |  | 17.2 |  |
| Prior allo-HSCT, % |  |  |  |  |  |  |  |
| No | 100.0 | 97.3 | 0.237 | 100.0 | 0.000 | 100.0 | 0.000 |
| Yes | 0.0 | 2.7 |  | 0.0 |  | 0.0 |  |
| Prior auto-HSCT, % |  |  |  |  |  |  |  |
| No | 55.9 | 66.8 | 0.225 | 55.9 | 0.000 | 55.9 | 0.000 |
| Yes | 44.1 | 33.2 |  | 44.1 |  | 44.1 |  |
| Bridging therapy, % |  |  |  |  |  |  |  |
| No | 8.6 | 41.4 | 0.819 | 47.8 | 0.967 | 58.0 | 1.23 |
| Yes | 91.4 | 58.6 |  | 52.2 |  | 42.0 |  |
| Number of prior lines of systemic therapy, recalculated per JULIET definition, % |  |  |  |  |  |  |  |
| 1 | 5.4 | 0.4 | 0.937 | 0.5 | 0.976 | 0.0 | 0.342 |
| 2 | 47.3 | 19.5 |  | 17.1 |  | 52.7 |  |
| 3 | 29.0 | 26.6 |  | 28.0 |  | 29.0 |  |
| 4‒6 | 18.3 | 43.4 |  | 46.3 |  | 18.3 |  |
| 7+ | 0.0 | 9.8 |  | 8.0 |  | 0.0 |  |
| Missing | 0.0 | 0.4 |  | 0.0 |  | 0.0 |  |
| Relapsed or refractory to last therapy, recalculated per JULIET definition, % |  |  |  |  |  |  |  |
| Refractory | 51.6 | 63.7 | 0.251 | 51.6 | 0.000 | 51.6 | 0.000 |
| Relapsed | 48.4 | 35.9 |  | 48.4 |  | 48.4 |  |
| Missing | 0.0 | 0.4 |  | 0.0 |  | 0.0 |  |
| Creatinine clearance prior to lymphodepleting chemotherapy, categorized per JULIET eligibility criteria, % |  |  |  |  |  |  |  |
| <60 mL/min | 0.0 | 19.1 | 0.688 | 22.1 | 0.754 | 0.0 | 0.000 |
| $\geq$60 mL/min | 100.0 | 80.9 |  | 77.9 |  | 100.0 |  |
| Left ventricle ejection fraction at screening, categorized per JULIET eligibility criteria, % |  |  |  |  |  |  |  |
| <45% | 0.0 | 1.6 | 0.178 | 1.5 | 0.173 | 0.0 | 0.000 |
| ≥45% | 100.0 | 98.4 |  | 98.5 |  | 100.0 |  |
| Pre-leukapheresis lymphocyte count (10^9^/L), categorized per JULIET eligibility criteria, % |  |  |  |  |  |  |  |
| <0.3 | 0.0 | 10.5 | 0.501 | 10.0 | 0.485 | 0.0 | 0.000 |
| ≥0.3 | 100.0 | 84.0 |  | 84.8 |  | 100.0 |  |
| Missing | 0.0 | 5.5 |  | 5.2 |  | 0.0 |  |
| Statistics |  |  |  |  |  |  |  |
| % of factors with SMD <0.2 |  | 26.7 |  | 60.0 |  | 86.7 |  |
| % of factors with SMD <0.1 |  | 6.7 |  | 46.7 |  | 86.7 |  |
| ABC: activated B cell; allo-HSCT: allogeneic hematopoietic stem cell transplantation; auto-HSCT: autologous hematopoietic stem cell transplantation; CNS: central nervous system; CR: complete response; DLBCL: diffuse large B-cell lymphoma; ECOG PS: Eastern Cooperative Oncology Group performance status; ESS: effective sample size; FL3B: follicular lymphoma grade 3B; GCB: germinal center B cell; HSCT: hematopoietic stem cell transplantation; IPI: International Prognostic Index; MAIC: matching-adjusted indirect comparison; N: sample size; OS: overall survival; PFS: progression-free survival; PMBCL: primary mediastinal B-cell lymphoma; SD: standard deviation; SMD: standardized mean difference; tFL: transformed follicular lymphoma. | | | | | | | |

Table S4 Comparison of clinical factors before and after MAIC of ORR in TRANSCEND and JULIET

| Clinical factor | Tisagenlecleucel (JULIET) efficacy analysis set | Liso-cel (TRANSCEND) DLBCL efficacy set  ORR | | | | | |
| --- | --- | --- | --- | --- | --- | --- | --- |
|  |  | Before MAIC (naive) | | After MAIC (primary) | | After MAIC (sensitivity) | |
|  | N=93 | N=256 | | ESS=164 | | ESS=37.3 | |
|  | Stat. | Stat. | SMD | Stat. | SMD | Stat. | SMD |
| IPI score, categorized per JULIET categorization, % |  |  |  |  |  |  |  |
| 0‒1 | 26.9 | 24.6 | 0.048 | 29.2 | 0.051 | 26.9 | 0.000 |
| 2‒5 | 73.1 | 74.6 |  | 70.8 |  | 73.1 |  |
| Missing | 0.0 | 0.8 |  | 0.0 |  | 0.0 |  |
| ECOG PS at screening, % |  |  |  |  |  |  |  |
| 0‒1 | 100.0 | 98.4 | 0.178 | 100.0 | 0.000 | 100.0 | 0.000 |
| 2 | 0.0 | 1.6 |  | 0.0 |  | 0.0 |  |
| Disease stage, % |  |  |  |  |  |  |  |
| I or II | 22.6 | 27.0 | 0.106 | 29.4 | 0.155 | 22.6 | 0.000 |
| III or IV | 77.4 | 72.3 |  | 70.6 |  | 77.4 |  |
| Missing | 0.0 | 0.8 |  | 0.0 |  | 0.0 |  |
| Secondary CNS lymphoma at time of treatment, % |  |  |  |  |  |  |  |
| No | 100.0 | 97.7 | 0.219 | 100.0 | 0.000 | 100.0 | 0.000 |
| Yes | 0.0 | 2.3 |  | 0.0 |  | 0.0 |  |
| Disease histology, % |  |  |  |  |  |  |  |
| DLBCL | 80.6 | 71.1 | 0.393 | 74.4 | 0.149 | 80.6 | 0.000 |
| DLBCL tFL | 19.4 | 22.3 |  | 25.6 |  | 19.4 |  |
| PMBCL | 0.0 | 5.5 |  | 0.0 |  | 0.0 |  |
| FL3B | 0.0 | 1.2 |  | 0.0 |  | 0.0 |  |
| Cell of origin, % |  |  |  |  |  |  |  |
| GCB | 53.8 | 44.1 | 0.615 | 47.3 | 0.587 | 53.8 | 0.000 |
| ABC | 43.0 | 28.1 |  | 31.3 |  | 43.0 |  |
| Unknown | 3.2 | 21.1 |  | 21.5 |  | 3.2 |  |
| Missing | 0.0 | 6.6 |  | 0.0 |  | 0.0 |  |
| Double or triple hit, % |  |  |  |  |  |  |  |
| Unknown | 37.6 | 29.3 | 0.217 | 26.2 | 0.276 | 37.6 | 0.000 |
| No | 45.2 | 55.9 |  | 58.2 |  | 45.2 |  |
| Yes | 17.2 | 14.8 |  | 15.5 |  | 17.2 |  |
| Prior allo-HSCT, % |  |  |  |  |  |  |  |
| No | 100.0 | 97.3 | 0.237 | 100.0 | 0.000 | 100.0 | 0.000 |
| Yes | 0.0 | 2.7 |  | 0.0 |  | 0.0 |  |
| Prior autho-HSCT, % |  |  |  |  |  |  |  |
| No | 55.9 | 66.8 | 0.225 | 55.9 | 0.000 | 55.9 | 0.000 |
| Yes | 44.1 | 33.2 |  | 44.1 |  | 44.1 |  |
| Bridging therapy, % |  |  |  |  |  |  |  |
| No | 8.6 | 41.4 | 0.819 | 43.2 | 0.860 | 58.0 | 1.230 |
| Yes | 91.4 | 58.6 |  | 56.8 |  | 42.0 |  |
| Number of prior lines of systemic therapy, recalculated per JULIET definition, % |  |  |  |  |  |  |  |
| 1 | 5.4 | 0.4 | 0.937 | 0.5 | 0.972 | 0.0 | 0.342 |
| 2 | 47.3 | 19.5 |  | 17.7 |  | 52.7 |  |
| 3 | 29.0 | 26.6 |  | 26.4 |  | 29.0 |  |
| 4‒6 | 18.3 | 43.4 |  | 49.0 |  | 18.3 |  |
| 7+ | 0.0 | 9.8 |  | 6.4 |  | 0.0 |  |
| Missing | 0.0 | 0.4 |  | 0.0 |  | 0.0 |  |
| Relapsed or refractory to last therapy, recalculated per JULIET definition, % |  |  |  |  |  |  |  |
| Refractory | 51.6 | 63.7 | 0.251 | 63.7 | 0.247 | 51.6 | 0.000 |
| Relapsed | 48.4 | 35.9 |  | 36.3 |  | 48.4 |  |
| Missing | 0.0 | 0.4 |  | 0.0 |  | 0.0 |  |
| Creatinine clearance prior to lymphodepleting chemotherapy, categorized per JULIET eligibility criteria, % |  |  |  |  |  |  |  |
| <60 mL/min | 0.0 | 19.1 | 0.688 | 0.0 | 0.000 | 0.0 | 0.000 |
| $\geq$60 mL/min | 100.0 | 80.9 |  | 100.0 |  | 100.0 |  |
| Left ventricle ejection fraction at screening, categorized per JULIET eligibility criteria, % |  |  |  |  |  |  |  |
| <45% | 0.0 | 1.6 | 0.178 | 1.4 | 0.169 | 0.0 | 0.000 |
| ≥45% | 100.0 | 98.4 |  | 98.6 |  | 100.0 |  |
| Pre-leukapheresis lymphocyte count (10^9^/L), categorized per JULIET eligibility criteria, % |  |  |  |  |  |  |  |
| <0.3 | 0.0 | 10.5 | 0.501 | 8.8 | 0.457 | 0.0 | 0.000 |
| ≥0.3 | 100.0 | 84.0 |  | 84.2 |  | 100.0 |  |
| Missing | 0.0 | 5.5 |  | 7.1 |  | 0.0 |  |
| Statistics |  |  |  |  |  |  |  |
| % of factors with SMD <0.2 |  | 26.7 |  | 60.0 |  | 86.7 |  |
| % of factors with SMD <0.1 |  | 6.7 |  | 40.0 |  | 86.7 |  |
| ABC: activated B cell; allo-HSCT: allogeneic hematopoietic stem cell transplantation; auto-HSCT: autologous hematopoietic stem cell transplantation; CNS: central nervous system; DLBCL: diffuse large B-cell lymphoma; ECOG PS: Eastern Cooperative Oncology Group performance status; ESS: effective sample size; FL3B: follicular lymphoma grade 3B; GCB: germinal center B cell; HSCT: hematopoietic stem cell transplantation; IPI: International Prognostic Index; MAIC: matching-adjusted indirect comparison; N: sample size; ORR: objective response rate; PMBCL: primary mediastinal B-cell lymphoma; SD: standard deviation; SMD: standardized mean difference; tFL: transformed follicular lymphoma. | | | | | | | |

Table S5 Comparison of clinical factors before and after MAIC for safety analysis in TRANSCEND and JULIET

| Clinical factor | Tisagenlecleucel (JULIET) safety set/full analysis set | Liso-cel (TRANSCEND) DLBCL treated set  safety analysis | | | |
| --- | --- | --- | --- | --- | --- |
|  |  | Before MAIC | | After MAIC | |
|  | N=111 | N=269 | | ESS=122.9 | |
|  | Stat. | Stat. | SMD | Stat. | SMD |
| Age, years |  |  |  |  |  |
| Mean (SD) | 53.9 (12.9) | 60.1 (13.3) | 0.467 | 59.1 (14.8) | 0.367 |
| ECOG PS at screening, % |  |  |  |  |  |
| 0 | 55.0 | 40.9 | 0.324 | 55.0 | 0.000 |
| 1 | 45.0 | 57.6 |  | 45.0 |  |
| 2 | 0.0 | 1.5 |  | 0.0 |  |
| Secondary CNS lymphoma at time of treatment, % |  |  |  |  |  |
| No | 100.0 | 97.4 | 0.231 | 100.0 | 0.000 |
| Yes | 0.0 | 2.6 |  | 0.0 |  |
| Prior HSCT, % |  |  |  |  |  |
| Allo-HSCT | 0.0 | 3.3 | 0.263 | 0.0 | 0.000 |
| Auto-HSCT | 48.6 | 33.5 | 0.312 | 21.0 | 0.605 |
| Number of prior lines of systemic therapy, recalculated per JULIET definition, % |  |  |  |  |  |
| 1 | 4.5 | 0.4 | 0.885 | 4.6 | 0.001 |
| 2 | 44.1 | 19.0 |  | 44.1 |  |
| 3 | 30.6 | 26.0 |  | 30.6 |  |
| 4‒6 | 20.7 | 43.9 |  | 20.7 |  |
| 7+ | 0.0 | 10.4 |  | 0.0 |  |
| Missing | 0.0 | 0.4 |  | 0.0 |  |
| Statistics |  |  |  |  |  |
| % of factors with SMD <0.2 |  | 0 |  | 66.7 |  |
| % of factors with SMD <0.1 |  | 0 |  | 66.7 |  |
| Allo-HSCT: allogenic hematologic stem cell transplant; auto-HSCT: autologous hematologic stem cell transplant; CNS: central nervous system; ECOG PS: Eastern Cooperative Oncology Group performance status; ESS: effective sample size; MAIC: matching-adjusted indirect treatment comparison; N: sample size; SD: standard deviation; SMD: standardized mean difference. | | | | | |
